# Using Wear Time for the Analysis of Consumer-Grade Wearables’ Data: Case Study Using Fitbit Data

**DOI:** 10.2196/46149

**Published:** 2025-03-21

**Authors:** Loubna Baroudi, Ronald Fredrick Zernicke, Muneesh Tewari, Noelle E Carlozzi, Sung Won Choi, Stephen M Cain

**Affiliations:** 1 Department of Mechanical Engineering, University of Michigan–Ann Arbor, 2505 Hayward St, Ann Arbor, MI, 48109, United States, 1 7342626353; 2 Department of Orthopedic Surgery, University of Michigan–Ann Arbor, Ann Arbor, MI, United States; 3 Exercise & Sport Science Initiative, University of Michigan–Ann Arbor, Ann Arbor, MI, United States; 4 Center for Computational Medicine and Bioinformatics, University of Michigan–Ann Arbor, Ann Arbor, MI, United States; 5 Department of Internal Medicine, University of Michigan–Ann Arbor, Ann Arbor, MI, United States; 6 Department of Biomedical Engineering, University of Michigan–Ann Arbor, Ann Arbor, MI, United States; 7 Veterans Administration Ann Arbor Healthcare System, University of Michigan–Ann Arbor, Ann Arbor, MI, United States; 8 Department of Physical Medicine and Rehabilitation, University of Michigan–Ann Arbor, Ann Arbor, MI, United States; 9 Department of Pediatrics, University of Michigan–Ann Arbor, Ann Arbor, MI, United States; 10 Department of Chemical and Biomedical Engineering, West Virginia University, Morgantown, WV, United States

**Keywords:** wear time, wearables, smartwatch, mobile health, physical activity, engagement, walking, dataset, wearable devices, reliability, behavior, caregiver, students, Fitbit, users

## Abstract

**Background:**

Consumer-grade wearables allow researchers to capture a representative picture of human behavior in the real world over extended periods. However, maintaining users’ engagement remains a challenge and can lead to a decrease in compliance (eg, wear time in the context of wearable sensors) over time (eg, “wearables’ abandonment”).

**Objective:**

In this work, we analyzed datasets from diverse populations (eg, caregivers for various health issues, college students, and pediatric oncology patients) to quantify the impact that wear time requirements can have on study results. We found evidence that emphasizes the need to account for participants’ wear time in the analysis of consumer-grade wearables data. In Aim 1, we demonstrate the sensitivity of parameter estimates to different data processing methods with respect to wear time. In Aim 2, we demonstrate that not all research questions necessitate the same wear time requirements; some parameter estimates are not sensitive to wear time.

**Methods:**

We analyzed 3 Fitbit datasets comprising 6 different clinical and healthy population samples. For Aim 1, we analyzed the sensitivity of average daily step count and average daily heart rate at the population sample and individual levels to different methods of defining “valid” days using wear time. For Aim 2, we evaluated whether some research questions can be answered with data from lower compliance population samples. We explored (1) the estimation of the average daily step count and (2) the estimation of the average heart rate while walking.

**Results:**

For Aim 1, we found that the changes in the population sample average daily step count could reach 2000 steps for different methods of analysis and were dependent on the wear time compliance of the sample. As expected, population samples with a low daily wear time (less than 15 hours of wear time per day) showed the most sensitivity to changes in methods of analysis. On the individual level, we observed that around 15% of individuals had a difference in step count higher than 1000 steps for 4 of the 6 population samples analyzed when using different data processing methods. Those individual differences were higher than 3000 steps for close to 5% of individuals across all population samples. Average daily heart rate appeared to be robust to changes in wear time. For Aim 2, we found that, for 5 population samples out of 6, around 11% of individuals had enough data for the estimation of average heart rate while walking but not for the estimation of their average daily step count.

**Conclusions:**

We leveraged datasets from diverse populations to demonstrate the direct relationship between parameter estimates from consumer-grade wearable devices and participants’ wear time. Our findings highlighted the importance of a thorough analysis of wear time when processing data from consumer-grade wearables to ensure the relevance and reliability of the associated findings.

## Introduction

Physical activity can be used to prevent and treat multifarious health issues. In the clinical setting and in research studies, the assessment of physical activity level is often carried out using self-report questionnaires. However, because self-reports are low in resolution, subjective, and often limited in scope, researchers have been looking for solutions to objectively quantify physical activity [1,2]. In the early 21st century, smartwatches and fitness trackers entered the consumer market [3]. These consumer-grade devices integrate a combination of sensors to measure key health metrics such as step count or heart rate. With the realization that fitness trackers can motivate users to exercise by providing them with real-time feedback, researchers and clinicians are now increasingly using these devices to monitor individuals [4-7]. In particular, Fitbit (Google Inc) smartwatches have imposed themselves in the clinical world due to their low price, high battery life, user-friendliness, and compatibility with most smartphones on the market. However, useful data can only be collected if participants are compliant and wear the watch.

With longitudinal studies, it can be challenging to maintain users’ engagement and compliance. It is common to observe what is called “wearables’ abandonment”; as the excitement from having a new gadget wears off, people start to wear the sensors less and less [8]. In the accelerometer research—where accelerometers are commonly used to monitor physical activity—researchers have established different methods to account for wear time in the data processing pipeline [9-12]. In both the Fitbit and the accelerometer literature, when wear time is considered, researchers use different thresholds on daily wear time or on step count to preprocess their data and extract valid days [13-16]. However, as wear time and activity levels vary for each study population, those thresholds might not always be generalizable or may be overly strict for some research questions. For instance, a very active population might need a higher step count threshold compared to a sedentary population. This suggests that researchers should evaluate whether their selected wear time requirements are appropriate for their study population and research question to ensure the validity of their data processing [17]. Researchers should also present a justification for their wear time requirements. Furthermore, as different research questions might require a different amount of data, the required amount of data will need to be adapted.

In this work, we aimed to further the understanding of the effects of compliance on parameter estimates. Our objective was to (Aim 1) quantify the sensitivity of calculations to different wear time requirements between and within different population samples and (Aim 2) demonstrate that not all research questions may require identical wear time requirements. We leveraged three Fitbit datasets from six different populations to illustrate this work: the estimation of the average (1) daily step count, (2) daily heart rate, and (3) average heart rate while walking. As the use of consumer-grade wearables is rapidly increasing, this work provides a thorough demonstration of the potential impact of wear time on essential parameter estimates in a diversity of populations (clinical and nonclinical) with varying degrees of compliance. This work provides quantitative evidence of the need to use wear time requirements in data analyses, specifically for study population samples with low compliance.

## Methods

### Description of Datasets

For this study, 3 Fitbit datasets from 6 different populations were used (Table 1).

**Table 1.** Dataset details.

Dataset	Caregivers			Students	Pediatric oncology	
	Hematopoietic cell transplantation	Huntington disease	Spinal cord injury		Caregivers	Patients
Sample size, n	30	21	19	2107	49	44
Study length, days	90	90	90	90	120	120
Fitbit type	Fitbit Inspire 2	Fitbit Inspire 2	Fitbit Inspire 2	Fitbit Charge	Fitbit Charge	Fitbit Charge
Fitbit wear instructions	Wear as much as possible	Wear as much as possible	Wear as much as possible	40 h/wk	Wear as much as possible	Wear as much as possible
Fitbit wear incentives	Fitbit given + US $1/day if some Fitbit OR survey data	Fitbit given + US $1/day if some Fitbit OR survey data	Fitbit given + US $1/day if some Fitbit OR survey data	Fitbit given	Fitbit given	Fitbit given

#### Three Different Caregiver Groups

The details of this study protocol can be found in Carlozzi et al [18]. Briefly, caregivers for persons with Huntington disease (HD), spinal cord injury (SCI), and hematopoietic cell transplantation (HCT) from different clinics at the University of Michigan were recruited between November 2020 and June 2021. This study’s objective was to evaluate a just-in-time adaptive intervention to promote caregivers’ self-care. This intervention leveraged the combination of Fitbit and survey data collected using an app (TBI-CareQOL). It is important to note that participants were reminded to sync their Fitbit data every Monday and Friday if they had not already done so.

#### College Students

The details of this study protocol can be found in Cislo et al [19]. Briefly, graduate and undergraduate students at the University of Michigan were recruited between September 2020 and December 2020 to study students’ mental health and physical activity during the COVID-19 pandemic. Different measures were collected using surveys, a smartphone app (Roadmap 2.0), and a Fitbit smartwatch.

#### Pediatric Oncology Caregivers and Patients

This dataset was collected in a study evaluating the use of a mobile health app (Roadmap 2.0) intervention for cancer caregivers and their patients [20]. Participants were recruited between September 2020 and September 2021 from the Adult and Pediatric Hematology and Oncology Units of Mott Children’s Hospital in Ann Arbor, MI.

### Data Analysis and Statistics

#### Compliance Levels for Each Population

We first compared the level of compliance and wear time for each population. To calculate wear time, we used the heart rate data from the Fitbit watch [13]. A heart rate value was registered by the watch every minute if the watch was worn. Thus, wear time was calculated as follows:


(1) $$\text{Wear time}=\frac{\text{\# Minutes of registered heart rate}}{\text{Total minutes}}$$

Total minutes depended on the time frame of interest. For instance, if we wanted to calculate wear time over 24 hours, the total number of minutes was 1440 minutes. Because our groups did not have a similar variance, and the sample sizes were largely different, the difference in wear time between groups was evaluated using a Kruskal-Wallis test [21]. If the omnibus test was significant, the Dunn test was used to evaluate pairwise differences [22]. Significance was evaluated at the .05 level.

#### Aim 1: Sensitivity of Parameter Estimates to Different Wear Time Requirements

For Aim 1, three different definitions of compliance were used to evaluate the impact on the evaluation of the average daily step count and average daily heart rate for the population and the individual. Both of these measures have been used to determine fitness levels in multifarious populations [20-22]. Compliance was expressed in the form of valid days, where a valid day corresponded to a day that met a certain criterion and was kept for analysis. The 3 different definitions of a valid day were as follows: (*None*) all days were considered valid, (*StepCount1000*) a day was valid if the step count registered for that day was greater than 1000, and (*WearTime80*) a day was valid if the wear time that day (24 hours) was greater than 80% (19.2 hours). For that last definition, wear time was calculated using equation (1). The definitions of a valid day *StepCount1000* and *WearTime80* were based on studies found in the literature [13,14]. These 2 definitions also offer different perspectives on wear time by leveraging 2 distinct data types (step count vs wear time, defined here using heart rate). We also added the definition *None* to demonstrate the impact of not including wear time in analyses, which is the case in a large number of studies that use consumer-grade wearables [17]. The average daily step count and heart rate were calculated for the population and each individual using these 3 different definitions. The code to extract participants’ wear time using Fitbit heart rate data and evaluate effects on step count is available on GitHub [23]. This repository includes the data preprocessing scripts and analysis code using an open-source Fitbit dataset.

#### Aim 2: Effect of Compliance on the Research Objective

**Figure 1.**
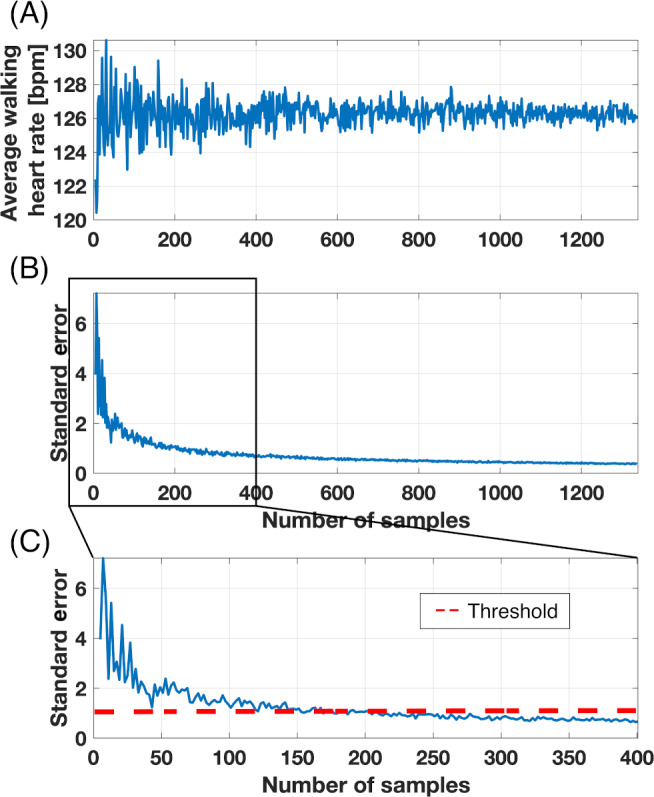
Evolution of average steps taken in a minute (A) and standard error (in steps) (B and C) with an increase in the number of samples for a representative subject. (C) is the expansion of (B) to visualize the number of samples necessary for this individual to obtain a standard error below 2. bpm: beats per minute.

For Aim 2, we examined the effect of compliance on the ability to address different research questions. We wanted to investigate whether some research questions can be answered, even when subject compliance is poor. Two research objectives were compared for this aim: (1) the evaluation of average daily step count and (2) the evaluation of average heart rate while walking. Heart rate while walking can be an indicator of cardiac health and exercise intensity [24-26]. To obtain heart rate while walking, we used Fitbit’s number of steps taken in a minute and isolated instances where that value was above 80 steps taken in a minute. That indicated a high likelihood for an individual to be on a walk [27]. Then, we isolated the values of heart rate corresponding to those instances that we named walking heart rate. To determine the minimal number of samples of walking heart rate (eg, the number of data points of heart rate corresponding to our definition of walking heart rate) needed to converge to a confident average, the standard error was calculated for an increasing random number of samples. A threshold of 1 bpm (beats per minute) was set for convergence (Figure 1). Once the minimum number of samples necessary for each individual was found, the number of days needed to get this amount of samples was extracted.

### Ethical Considerations

All datasets were generated by teams at Michigan Medicine at the University of Michigan, and the different data collection protocols were each approved by the University of Michigan institutional review board, under the review numbers HUM00186436, HUM00176584, HUM00184455, and HUM00185391. All participants provided informed consent prior to their participation in the study. The details of the compensation for each study are provided in Table 1.

## Results

### Wear Time

**Figure 2.**
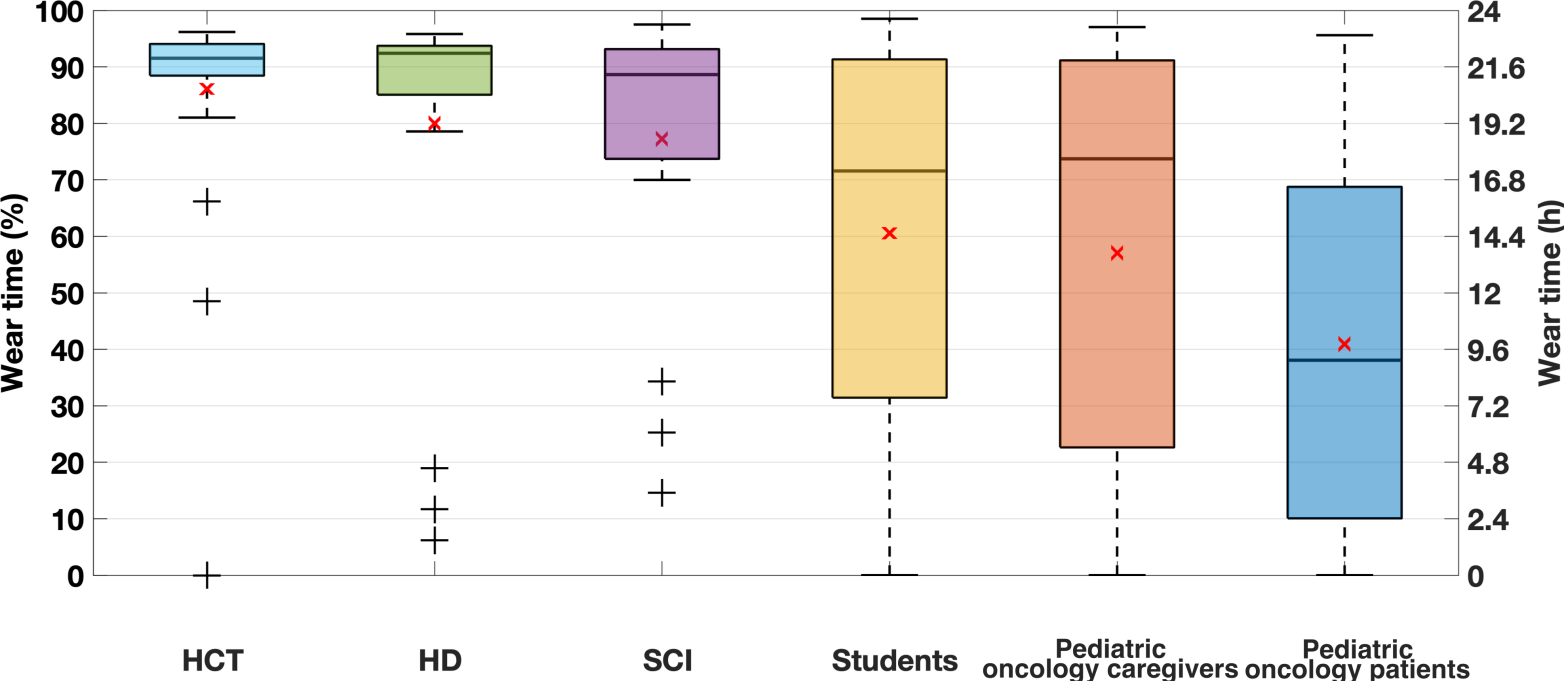
Distribution of average daily wear time in percentages (left) and hours (right) for each recruited sample. The pediatric oncology patients and caregivers population, as well as the student population, had participants that were given a Fitbit and never wore it. As such, there are instances with 0 hours of daily wear time in the distributions. HCT: hematopoietic cell transplantation; HD: Huntington disease; SCI: spinal cord injury.

The average wear time of the device varied by population (Figure 2). The most compliant sample was the HCT caregivers with an average daily wear time of 20.8 hours. The least compliant sample was the pediatric oncology patients with an average daily wear time of 9.8 hours. Differences were also observed in the spread of the data. For the pediatric oncology patients and caregivers and the student sample, the distribution went from 0% to above 90% of wear time. A 0% wear time indicated that participants received the Fitbit but never wore it.

There were significant differences in the average wear time between populations (Table 2). Notably, the pediatric oncology patients had significantly lower wear time than all other studied samples except for the pediatric oncology caregivers. The HCT caregivers also had significantly higher wear time than the other groups, except for the SCI and HD caregivers.

A decrease was observed in monthly wear time for all recruited groups. The average decrease was a little more than 10%, with the highest decrease recorded for the pediatric oncology patients (−19%) and the lowest decrease recorded for the HCT caregivers (−8%).

**Table 2.** Model coefficients and significance from post hoc test evaluating the difference in wear time between samples.

	HCT^a^	HD^b^	SCI^c^	Students	Pediatric oncology caregivers
HD	0.52	—^d^	—	—	—
SCI	1.04	0.5	—	—	—
Students	4.23^e^	2.88	2.04	—	—
Pediatric oncology caregivers	3.68^e^	2.7	2.02	0.52	—
Pediatric oncology patients	5.83^e^	4.65^e^	3.91^e^	3.96^e^	2.54

a HCT: hematopoietic cell transplantation.

b HD: Huntington disease.

c SCI: spinal cord injury.

d Not applicable.

e *P*<.001.

### Aim 1

#### Population Level

**Figure 3.**
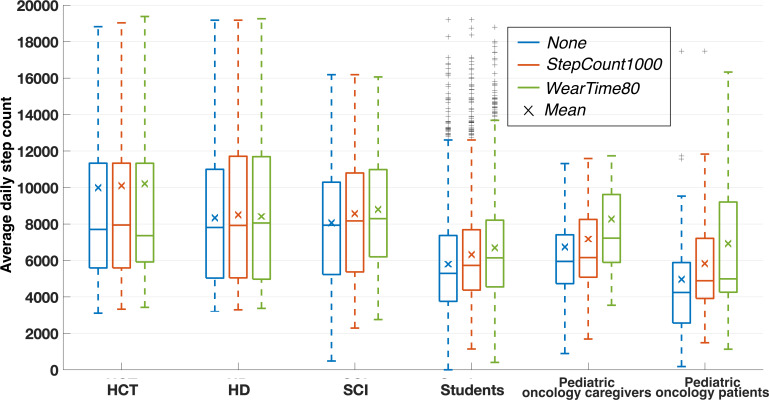
Average daily step count across samples for the different definitions of a valid day. The effect of these different definitions was dependent on the sample, going from less than 80 steps for the HD sample to almost 2000 steps for the pediatric oncology patients. HCT: hematopoietic cell transplantation; HD: Huntington disease; SCI: spinal cord injury.

First, we observed the changes in the sample average daily step count for the 3 chosen definitions of a valid day (Figure 3). The average number of valid days decreased between 15% and 33% from the definition *None* to *WearTime80*. The decrease from *None* to *StepCount1000* was around 12% for the SCI caregivers and the pediatric oncology patients and close to 0% for the other samples. There was also a decrease from *StepCount1000* to *WearTime80* from 12% to 21%. The change in average daily step count was dependent on the sample. HCT and HD caregivers both showed a difference smaller than 15 steps between *None* and *WearTime80*. On the other hand, the pediatric oncology patients showed a difference of almost 2000 steps between *None* and *WearTime80*. The sample sizes decreased from *None* to *WearTime80* for the pediatric oncology patients, caregivers, and students (n=6, −14%; n=10, −20%; and n=7, −8%; respectively).

We also analyzed changes in the sample average daily heart rate for the 3 definitions. We found only small differences in average daily heart rate for all samples. The student sample showed the largest difference of 3 bpm between *None* and *WearTime80*.

#### Individual Level

**Figure 4.**
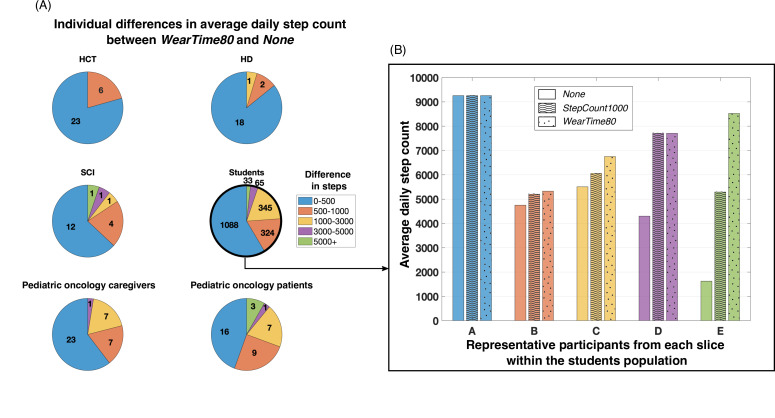
(A) Individual differences in average daily step count calculated using *None* versus *WearTime80*. For example, 21% (n=6) of the HCT sample had a difference in average daily step count between 500 and 999 steps when calculations were made using *WearTime80* versus *None*. The grouping of the step differences was inspired by the physical activity level categories presented by Tudor-Locke and Bassett [28]. (B) Five representative participants’ average daily step count from the different slices of difference in step count, using the 3 definitions of a valid day. For example, participant E was representative of students who had a difference in average daily step count larger than 5000 when calculated with *WearTime80* compared to *None*. HCT: hematopoietic cell transplantation; HD: Huntington disease; SCI: spinal cord injury.

We assessed the changes in each individual’s average daily step count (Figure 4). We binned individuals based on the average daily step count difference between *None* and *WearTime80,* since those definitions were the least and most stringent, respectively (Figure 4A). For all samples, the majority of individuals had a difference in average daily step count between 0 and 500 steps. The HCT caregivers had the least differences in individual steps, followed by the HD caregivers. Approximately, a quarter of the SCI caregivers, pediatric oncology patients and caregivers, and students had a step count difference higher than 1000. By taking a closer look at the individuals in each bin for a group (Figure 4B), we saw that participants like participant A did not display any difference in average daily step count between each definition. However, participants like participant E showed a difference higher than 3000 steps between each definition, with that difference reaching almost 7000 steps between *None* and *WearTime80*.

Little to no variation was observed within participants for the average daily heart rate between *None* and *WearTime80*. Only 3% (n=1) of the pediatric oncology patient participants had a difference between 5 and 10 bpm, and less than 1% (n=21) of students had a difference between 10 and 15 bpm.

### Aim 2

**Figure 5.**
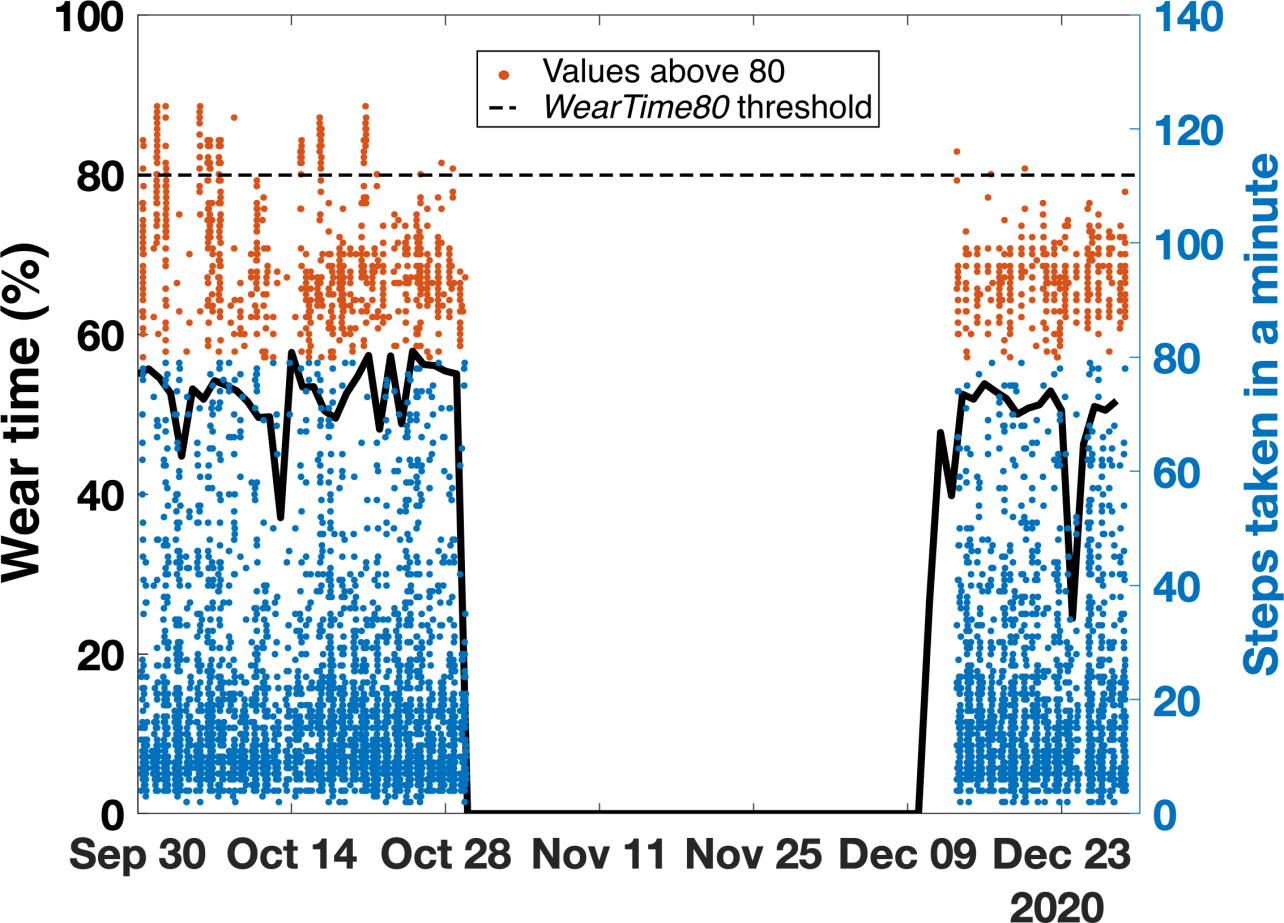
Wear time (black line, left axis) and steps taken in a minute (blue and orange dots, right axis) over time for an individual from the student sample. Average daily step count using *WearTime80* with this participant was not possible as no days presented a wear time larger than 80%. However, there was a sufficient number of samples to confidently estimate walking heart rate based on the number of samples of steps taken in a minute that was above 80.

On average, across all population samples, 304 samples (minutes of walking) were needed to obtain an estimate of the walking heart rate with a standard error of 1 bpm. For most individuals, the minimal number of samples was reached in under 26 days of data collection. For the pediatric oncology caregivers, 1 participant did not have enough samples to obtain a confident estimate of the walking heart rate, but an estimate of that caregiver’s average daily step count using *WearTime80* was obtained. On the other hand, 5 (17%) HCT, 3 (14%) HD, 2 (11%) SCI, 186 (10%) students, and 3 (7%) of the pediatric oncology patients did not have any valid days to calculate their average daily step count but presented enough samples to confidently obtain their walking heart rate. Figure 5 illustrates an individual’s data from the student sample within that 10%. That individual wore the Fitbit at the beginning and the end of the study for around 50% of the day. That student had 1314 samples of steps taken in a minute—enough to estimate walking heart rate—while presenting no days with a wear time higher than 80%.

## Discussion

### Principal Results

The purpose of this study was to quantify how wear time could impact parameter estimates in studies using consumer-grade wearables by using real-world samples with varying participant compliance. This work is distinguished from the literature by demonstrating the conditional nature of wear time’s influence in consumer-grade wearables’ data, which varies significantly across research samples and research questions. By leveraging a broad array of datasets, including vulnerable patient populations, we showed evidence of the nuanced effect of wear time, highlighting its critical role in research using wearables, especially in samples with inconsistent wear patterns such as the pediatric oncology caregiver and patient participants used in this study. It is important to note that the pediatric oncology patients’ ages ranged from 5 to 20 years, with a mean of 11.9 years. The young age combined with illness for these subjects likely explained this population sample’s low level of wear time with large variability.

**Figure 6.**
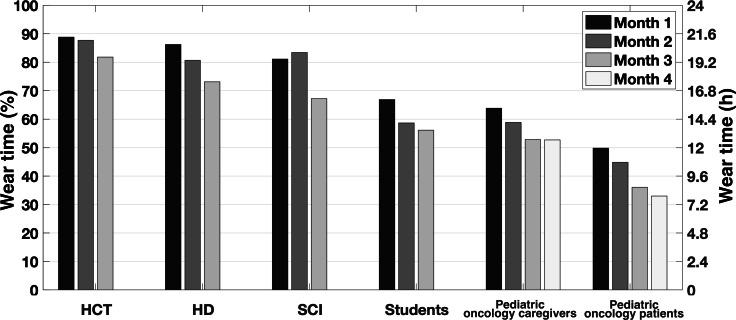
Monthly average daily wear time in percentages (left) and hours (right). We observed a monthly decrease in wear time for all groups symptomatic of the phenomenon of wearables’ abandonment. The pediatric oncology dataset was collected over 120 days versus 90 days for the other datasets. HCT: hematopoietic cell transplantation; HD: Huntington disease; SCI: spinal cord injury.

Unsurprisingly, implementing different wear time requirements to define a valid day can significantly impact parameter estimates, especially in samples with low compliance. Wear time compliance for the HD and HCT caregivers was high (Figure 6); therefore, the estimates of the samples’ average daily step counts were minimally affected by different definitions of a valid day (Figure 3). In comparison, the pediatric oncology patient sample had low wear time compliance (Figure 6); therefore, the estimate of the sample average daily step count was significantly impacted by different definitions of a valid day (Figure 3). Importantly, our analysis quantifies these differences so that other researchers can better understand the potential implications of different valid day definitions. The definition of a valid day had almost no impact on estimates of the average daily heart rate across samples. This also is not surprising as heart rate is a more stable measure that does not necessarily correlate with wear time; any biases from missing data are averaged out.

A significant novelty in this work is demonstrating the flexibility of data usage in real-world settings (ie, outside of the hospital). In fact, we showed that even if an individual’s data were not sufficient to answer a specific research question, those data could potentially still be leveraged toward a different analysis (eg, for the estimate of walking heart rate as shown in this study). In particular, the variations in the research question time frame (eg, daily, nighttime, and weekly) can lead to different levels of required compliance. For example, to accurately estimate the average number of steps per day, we need a sufficient number of days with high wear time. However, for the example we used in Aim 2—the estimation of walking heart rate—we need a sufficient number of steps taken in a bout of walking by an individual. As such, we show in our example how an individual can have a large amount of walking data throughout an entire study while not having enough individual days with high wear time (Figure 5). These findings suggest that even samples with low compliance may still be useful for answering research questions. In this case, even a day with low wear time (ie, not a valid day) may contain several valid walks useful for estimating average walking heart rate.

### Secondary Findings

We also found that depending on whether one is interested in studying a population as a whole or the individuals within a population, the impact of wear time differed. For instance, in the student population, we observed only a small change in the estimated average daily step count in the population with the different methods of analysis (Figure 3). Depending on the objective of a study, that change could be negligible. However, if research is conducted to design a physical activity intervention or to evaluate the efficacy of an intervention, for example, the processing of an individual’s data without the inclusion of wear time could lead to critical errors (Figure 4). In particular, if one considers the standard classification of participants into levels of physical activity (0‐5000 daily steps: sedentary; 5000‐7500: low active; 7500‐10,000: somewhat active; 10,000+: highly active) [28], those errors can induce a misclassification. Ultimately, the intervention design or the evaluation of the intervention could be erroneous.

We quantified the phenomenon of wearables’ abandonment within our different populations (Figure 6). That phenomenon can potentially create a bias in results over time. For example, a monthly decrease in the population sample level of physical activity can be explained by an actual decline in people’s movements or by the fact that participants were not wearing their sensors as much. An appropriate quantification of wear time in the analysis can potentially remove that bias.

### Comparison With Prior Work

While the literature on accelerometer usage presents various methodologies for accounting for wear time, it is essential to acknowledge that raw acceleration data or activity counts, typical outputs from accelerometers, are not available from consumer-grade wearables. Therefore, the strategies for considering wear time in the context of these devices necessitate their own approach. Consumer-grade wearables often offer summarized or processed data, which requires tailored methods for wear time analysis to ensure accurate and meaningful interpretation in research contexts. In general, research data on the analysis of wear time and compliance with consumer-grade wearables are sparse in the literature. Often, studies using consumer-grade wearables, such as smartwatches, rely on threshold-based methodologies for processing wear time [13-16]. However, these thresholds are often implemented without sufficient justification based on the specific dataset being analyzed and the research objectives.

Claudel et al [17] developed a novel method to calculate wear time and determine a valid assessment day. Their proposed method calculated wear time using heart rate detection, step count, and the range of heart rate values. A valid day was defined as a day with more than 10 hours of wear time, with the removal of sleeping hours (from 11 PM to 5 AM). Claudel and colleagues present a structured and detailed methodology that can potentially be replicated for other studies. It highlights the importance of nighttime in wear time calculations and how researchers can conduct a sensitivity analysis to determine the impact of different nighttime limits on the outcome variables. Our study also demonstrates the potential impact of different calculations of wear time on parameter estimates. Both studies illustrate the importance of considering how the definition of a valid day may impact sample parameter estimates.

Studies have explored the factors that are related to compliance levels [13]. The NetHealth study [13] collected data from a student population using Fitbit devices and showed that some personality traits (eg, extraversion and openness) and Fitbit metrics (eg, minutes asleep and sedentary minutes) were related to compliance levels. Understanding the underlying reasons that may lead an individual to be more or less compliant can be useful in improving the overall wear time levels in these types of studies.

Another, perhaps more, direct way to increase compliance is through the use of incentives. There is a range of incentive models one can use to maintain compliance in longitudinal studies [29]. Although improving compliance allows for parameter estimates to be less sensitive to wear time, it is difficult to imagine a “one size fits all” incentive model that would work on every single participant of a study. Thus, the analysis of wear time is likely to remain a crucial step in the analysis of consumer-grade wearables data to ensure accurate parameter estimates.

### Limitations

In the accelerometer literature, raw outputs from the sensors are most often used to determine wear time [10,11], which removes a layer of potential error. The outputs provided by Fitbit are processed (as opposed to raw) with unknown algorithms. Here, we assumed those outputs, and in particular minute-by-minute heart rate, were reliable and could be used to accurately estimate wear time. Claudel et al [17] formulated a more complex wear time calculation algorithm fusing both heart rate detection and heart rate values revealing a potential overestimation of wear time when using heart rate detection only. Determining the most accurate estimate of wear time was beyond the scope of this study. However, further research should be conducted to validate methods for the calculation of wear time using the processed outputs of consumer-grade wearables as it may influence parameter estimates.

We assumed in Aim 2 that wear time was random. In other words, we assumed that participants did not choose a particular moment in their day to wear the device, which would bias the estimates of walking heart rate. However, this does not influence the conclusion of Aim 2, as we primarily focused on the amount of data needed to estimate a parameter with confidence, and not whether we could accurately estimate true walking heart rate.

It is important to note that the different datasets we used came from 2 different Fitbit devices (Fitbit Charge and Fitbit Inspire 2). It is possible that the versions of software and hardware are different between these 2 devices. Since the software information is proprietary information, we do not know whether the model differences impacted the used outputs. However, we expect and assume that the measurements between the 2 models are comparable and did not have a significant impact on our analysis.

Additionally, although we analyzed samples from different populations, our results may not translate to similar populations. First, each population sample was given a different set of instructions, which influenced their wear time. Second, Fitbit data syncing can be difficult and depends on the software used as well as the Fitbit model itself. Thus, the amount of data retrieved for the same population sample can be variable from one study to another. Finally, the different datasets used in this study were all collected during the COVID-19 pandemic, which may have affected the health-related behaviors of participants. We recommend that researchers conduct an analysis of wear time systematically when using sensing technologies to derive conclusions.

### Conclusions

One of the major benefits of leveraging fitness trackers is the potential to gain a unique and objective insight into an individual’s natural behavior. However, if the analysis of wear time is not appropriately integrated into the data processing pipeline, that purported insight can become biased and imprecise, driving the results away from reality. Herein, we presented evidence of the critical impact of wear time on parameter estimates and how that impact may vary depending on the population sample, research question, study design (ie, incentives), and participant compliance. Future research could significantly benefit from our findings and methodological examples to incorporate the following recommendations: first, it is essential to conduct a thorough analysis of the wear time of the population in any given study that uses wearables, with particular attention to the data available when using consumer-grade wearables; second, it is equally important to systematically document and provide comprehensive details on that analysis. Our work provides quantitative data on the impact that different valid day definitions can have on sample parameter estimates in samples with varying wear time compliance.

## Abbreviations

bpm: beats per minute
HCT: hematopoietic cell transplantation
HD: Huntington disease
SCI: spinal cord injury
